# Swine Interferon-Inducible Transmembrane Proteins Potently Inhibit African Swine Fever Virus Replication

**DOI:** 10.3389/fimmu.2022.827709

**Published:** 2022-03-25

**Authors:** Siqi Cai, Zezhong Zheng, JiaoJiao Cheng, Lintao Zhong, Ran Shao, Feiyan Zheng, Zhiying Lai, Jiajun Ou, Liang Xu, Pei Zhou, Gang Lu, Guihong Zhang

**Affiliations:** ^1^ Guangdong Provincial Key Laboratory of Zoonosis Prevention and Control, College of Veterinary Medicine, South China Agricultural University, Guangzhou, China; ^2^ African Swine Fever Regional Laboratory of China (Guangzhou), Guangzhou, China; ^3^ Maoming Branch, Guangdong Laboratory for Lingnan Modern Agriculture, Maoming, China; ^4^ Key Laboratory of Animal Vaccine Development, Ministry of Agriculture and Rural Affairs, Guangzhou, China; ^5^ Research Center for African Swine Fever Prevention and Control, South China Agricultural University, Guangzhou, China

**Keywords:** swine, interferon-inducible transmembrane protein, restriction, African swine fever virus, antiviral effect

## Abstract

African swine fever virus (ASFV) causes an acute, hemorrhagic, and highly contagious disease in domestic swine, leading to significant economic losses to the global porcine industry. Restriction factors of innate immunity play a critical in host antiviral action. However, function of swine restriction factors of innate immunity on ASFV has been seldomly investigated. In this study, we determined five homologues of swine interferon-induced transmembrane proteins (SwIFITM [named SwIFITM1a, -1b, -2, -3, and -5]), and we found that they all exhibit potent antiviral activity against ASFV. Expression profile analysis indicated that these SwIFITMs are constitutively expressed in most porcine tissues. Whether infected with ASFV or treated with swine interferon, the expression levels of SwIFITMs were induced *in vitro*. The subcellular localization of SwIFITMs was similar to that of their human homologues. SwIFITM1a and -1b localized to the plasma membrane, SwIFITM2 and -3 focused on the cytoplasm and the perinuclear region, while SwIFITM5 accumulated in the cell surface and cytoplasm. The overexpression of SwIFITM1a, -1b, -2, -3, or -5 could significantly inhibit ASFV replication in Vero cells, whereas knockdown of these genes could enhance ASFV replication in PAMs. We blocked the constitutive expression of endogenous IFITMs in Vero cells using a CRISPR-Cas9 system and then infected them with ASFV. The results indicated that the knockout of endogenous IFITMs could enhance ASFV replication. Finally, we expressed five SwIFITMs in knockout Vero cell lines and then challenged them with ASFV. The results showed that all of the SwIFITMs had a strong antiviral effect on ASFV. This research will further expand the understanding of the anti-ASFV activity of porcine IFITMs.

## Introduction

Interferons (IFNs) play an essential role in host defense against viral infections by inducing the expression of a variety of IFN-stimulated genes (ISGs) that can activate host antiviral immunity. interferon-induced transmembrane proteins (IFITMs) are a family of small transmembrane proteins that are critical ISG products. Compelling evidence has indicated that IFITMs can establish an innate immune state to eliminate pathogens efficiently (1, 2). IFITMs are known to inhibit the entry of a wide variety of RNA viruses, especially enveloped viruses such as the Ebola virus, influenza A virus (IAV), West Nile virus, hepatitis C virus, and dengue virus (1–6). However, little is known about the IFITM-mediated antiviral impact on DNA viruses. One recent study found that IFITM1 could inhibit *Rana grylio* virus (7), IFITM3 protein restricts cell infection by lyssaviruses to a similar level as its human orthologue (8), and human IFITMs have been identified as restriction factors for African swine fever virus (ASFV) (9).

IFITM proteins can impede broad-spectrum viral infection through various mechanisms. It is generally believed that IFITMs can block viral entry by suppressing viral membrane fusion (10). Additionally, some findings have indicated that IFITMs might also inhibit viral gene expression and viral protein synthesis and thereby impair viral replication. IFITMs may incorporate into virions during viral assembly and thus reduce the infectivity of nascent virions. The precise inhibitory mechanisms of IFITMs on viral infection and replication still require further exploration (11, 12).

To date, five swine IFITMs (SwIFITMs) have been found. These are located on chromosome 2, and they are referred to as SwIFITM1, -2, -3, -5, and -10. For SwIFITM1, there are two homologues differing in their N- and C-terminals, and these are named SwIFITM1a and -1b (13). Although IFITM5 is expressed mainly in osteoblasts and is required for bone mineralization (14), one study found that murine IFITM5 does not restrict IAV, but it efficiently inhibits filovirus entry (15). Few studies on IFITM10 have been reported, and its function remains largely unknown (14). IFITMs are widely distributed in cells, but there are great differences in the distribution and expression of their different subtypes (16). The differences in the subcellular localization and organelle distribution of SwIFITMs leads to the selectivity of their inhibitory effects on different viruses; that is, the sensitivity of a particular virus to different members of the family is different (17).

ASFV is the only member of the *Asfarviridae* family, and it causes an acute, hemorrhagic, and highly contagious disease affecting domestic swine. This often results in serious economic losses in many countries, especially China, due to the mortality rate of acute infection being close to 100% (18, 19). Currently, no commercial antiviral drugs are available for its control and effective treatment. SwIFITMs are a group of antiviral restriction factors that may target cell entry, gene duplication and transcription, protein translation, and virus assembly in the virus-replication cycle, which means that it may be possible to develop them as antiviral drugs or vaccine targets. In this study, we examined whether the SwIFITM family of proteins can affect ASFV infection.

## Materials and Methods

### Cells and Viruses

Human embryonic kidney cells (HEK-293T), Porcine Kidney-15 cells (PK-15), and Vero cells (ATCC CCL-81) were cultured in Dulbecco’s modified Eagle’s medium (DMEM; Life Technologies) supplemented with 10% fetal bovine serum (FBS; BI). Porcine alveolar macrophages (PAMs) were cultured in RPMI 1640 medium (Gibco) supplemented with 10% FBS. The GZ201801 strain of ASFV (GenBank: MT496893.1) was obtained from the Research Center for African Swine Fever Prevention and Control, South China Agricultural University (Guangzhou, China), which was a highly pathogenic strain belonging to p72 genotype II. Experiments involving ASFV were standardized according to the Laboratory Biosafety Manual and strictly performed in the P3 biosafety laboratory.

### Real-Time Quantitative PCR (RT-qPCR)

Specific primers and probes were designed based on the genetic sequences of swine IFITM1a, -1b, -2, -3, -5, β-actin, and Mx1, as well as ASFV p72 (**Table 1**). RT-qPCR was performed to define the relative mRNA expression of IFITMs and ASFV using a LightCycler 480 (Roche, Basel, Switzerland). Cells were treated with RNAiso Plus (Takara, Dalian, China) to extract total RNA, which was reversed transcribed into cDNA using the HiScript Q RT SuperMix for qPCR (Vazyme, Nanjing, China). RT-qPCR was performed using the TB Green Premix Ex Taq II kit (Takara, Dalian, China) or the AceQ qPCR Probe Master Mix kit (Vazyme, Nanjing, China) according to the manufacturer’s instructions. Relative fold changes in gene expression were normalized against β-actin using the 2^−ΔΔCt^ threshold method.

**Table 1:** Primers used for gene cloning and RT-qPCR detection.

Gene	Primer sequence (5′–3′)	Purpose
SwIFITM1a	F: ATGATCAAGAGCCAGCACG	Amplification of SwIFITM1a
	R: CTAGTAGCCTCTGTTACTCTTTGC	
SwIFITM1b	F: ATGCTCAGGGAGGAGCAC	Amplification of SwIFITM1b
	R: CTAGTAGCCTCTGTTACTCTTTGC	
SwIFITM2	F: ATGAACTGCGCTTCCC	Amplification of SwIFITM2
	R: CTAGTAGCCTCTGTTACTCT	
SwIFITM3	F: ATGAACTGCGCTTCCCAG	Amplification of SwIFITM3
	R: CTAGTAGCCTCTGTAATCCTTTATG	
SwIFITM5	F: ATGAGCTCACCCCCAGAC	Amplification of SwIFITM5
	R: TCAGTCATAGTCTGAGTCGTCGAA	
Sw-β-actin	F: CTGAACCCCAAAGCCAACCGT	RT-qPCR detection of swine β-actin
	R: TTCTCCTTGATGTCCCGCACG	
Sw-Mx1	F: TCTGTAAGCAGGAGACCATCAACT	RT-qPCR detection of swine Mx1
	R: TTTCTCGCCACGTCCACTATC	
SwIFITM1a	F: GGAGCCACTGTTCTTCTG	RT-qPCR detection of SwIFITM1a
	R: TAGCCTCTGTTACTCTTTGC	
SwIFITM1b	F: CGTCGCTCTTCTGGTGTT	RT-qPCR detection of SwIFITM1b
	R: GTAGCCTCTGTTACTCTTTGC	
SwIFITM2	F: CATTCTGACCATCGGAGCCA	RT-qPCR detection of SwIFITM2
	R: TTTGCGCGCTCTAACATCTG	
SwIFITM3	F: TGCGTTCATCATCGTTTGCAC	RT-qPCR detection of SwIFITM3
	R: TATGAGCTGCAGAACTGCTTGG	
SwIFITM5	F: CCTCTACCTGAACCTGTG	RT-qPCR detection of SwIFITM5
	R: CAGCCACCTTCTGATCTC	
ASFV p72	F: ATAGAGATACAGCTCTTCCAG	RT-qPCR detection of ASFV
	R: GTATGTAAGAGCTGCAGAAC	
	P: FAM-TATCGATAAGATTGAT-MGB	
siRNA1a,1b,2	CTGTTCTTCTGGTGTTTGT	siRNA targeting SwIFITM1a, -1b and -2
siRNA3	GCGTTCATCATCGTTTGCA	siRNA targeting SwIFITM3
siRNA5	CGCTGGCTTACTCCATCAA	siRNA targeting SwIFITM5
Flag-tagged SwIFITM1a	F:CGGGGTACCGCCACCATGGATTACAAGGATGACGACGATAAGGGATCCATGATCAAGAGCCAGCACG	Amplification of FLAG-tagged SwIFITM1a
	R:CCGGAATTCCTAGTAGCCTCTGTTACTCTTTGC	
Flag-tagged SwIFITM1b	F:CGGGGTACCGCCACCATGGATTACAAGGATGACGACGATAAGGGATCCATGCTCAGGGAGGAGCAC	Amplification of FLAG-tagged SwIFITM1b
	R:CCGGAATTCCTAGTAGCCTCTGTTACTCTTTGC	
Flag-tagged SwIFITM2	F:CGGGGTACCGCCACCATGGATTACAAGGATGACGACGATAAGGGATCCATGAACTGCGCTTCCC	Amplification of FLAG-tagged SwIFITM2
	R:CCGGAATTCCTAGTAGCCTCTGTTACTCT	
Flag-tagged SwIFITM3	F:CGGGGTACCGCCACCATGGATTACAAGGATGACGACGATAAGGGATCCATGAACTGCGCTTCCCAG	Amplification of FLAG-tagged SwIFITM3
	R:CCGGAATTCCTAGTAGCCTCTGTAATCCTTTATG	
Flag-tagged SwIFITM5	F:CGGGGTACCGCCACCATGGATTACAAGGATGACGACGATAAGGGATCCCCTCTACCTGAACCTGTG	Amplification of FLAG-tagged SwIFITM5
	R:CCGGAATTCTCAGTCATAGTCTGAGTCGTCGAA	
sgRNA-1	GATGGTTGGCGATGTGACCG	sgRNA targeting exon 1 of csIFITM1
sgRNA-3	GTGGTGAGTGCAATCGTCACA	sgRNA targeting exon 1 of csIFITM3
sgRNA-5	GGACACCGCGTATCCCCGCG	sgRNA targeting exon 1 of csIFITM5
csIFITM1	F: TCAACTGGTGCTGCCTGGG	Detection of knock out of csIFITM1
	R: ATCACAAGCACGTGCACTTTA	
csIFITM3	F: AGAGCAATTCTCCCTACAGC	Detection of knock out of csIFITM3
	R: CGCCCTTTCACAGAACTACTG	
csIFITM5	F: AGAGACGGCGCTGGAACCCATGGA	Detection of knock out of csIFITM5
	R: CCTCCCAATGACCTCTGTGC	

F, Forward; R, Reverse; P, Probe.

### Construction and Transfection of Plasmids and siRNAs

FLAG-tagged swine IFITM1a, -1b, -2, -3, and -5 were inserted into the eukaryotic expression vector pEF6/Myc-His (Life Technologies) to generate pEF-SwIFITMs. Small interfering RNAs (siRNAs) named siIFITM1a,1b,2 targeting SwIFITM1a, -1b and -2, two different siRNAs named siIFITM3 and siIFITM5 targeting SwIFITM3 and -5, respectively, and a negative-control nontargeting siRNA (siNC) were designed with the siRNA Designer website (Ribobio, Guangzhou, China). The plasmids and siRNAs were respectively transfected into Vero cells and PAMs using Lipo3000 transfection reagent (Thermo Fisher Scientific, Waltham, MA, USA) according to the manufacturer’s instructions.

### Establishment of Knockout Cell Lines

To make lentivirus, the lentiviral transfer plasmid lentiCRISPRv2-DsRed2 containing expression cassettes of hSpCas9 and the chimeric guide RNA modified from lentiCRISPRv2 (Addgene 52961) must be co-transfected into HEK293T cells with the packaging plasmid pMD2.G (Addgene 12259) and VSV-G envelope expressing plasmid psPAX2 (Addgene 12260) (20). Three different small guide RNAs (sgRNAs) targeting the *Chlorocebus sabaeus* IFITMs (csIFITMs) designed with the sgRNA Designer website (https://zlab.bio/guide-design-resources) were inserted into the lentiviral vector lentiCRISPRv2-DsRed2 to generate lentiCRISPRv2-DsRed2-csIFITMs. The lentiviruses were created by 0.5 µg pMD2.G, 0.5 µg psPAX2, 1 µg empty lentiCRISPRv2-DsRed2, or lentiCRISPRv2-DsRed2-csIFITMs, which were co-transfected into HEK-293T cells using Lipo3000 transfection reagent with Opti-MEM (Life Technologies) in six-well plates. Lentiviruses were collected in the cell supernatant 48 h after transfection by brief centrifugation at 1500 rpm for 15 min and 0.45-µM filtering and used to infect Vero cells seeded in the six-well plates. The positive cells were selected by the limiting dilution assay and fluorescence screening, and these continued to be cultivated in 96-well plates, generating single-cell clones (control or csIFITMs^−^ Vero cells) 72 h after infection (hpi). The knockout of csIFITMs was determined by Sanger’s sequencing validation or western blot (WB).

### Confocal Immunofluorescence Microscopy

The PK-15 cells were transfected with swine IFITM expression constructs in glass-bottomed coverslips (NEST Biotechnology, Wuxi, China) for 24 h. Then, the cells were fixed with 4% (vol/vol) paraformaldehyde at 4°C for 30 min. Following cell fixation, QuickBlock Blocking Buffer for Immunol Staining (Beyotime, Shanghai, China) was used. All the samples were stained with ANTI-FLAG monoclonal antibody (Sigma-Aldrich) to detect IFITM proteins and anti-LAMP1 monoclonal antibody (Abcam; ab25245) to detect endosomes, and they were then incubated with Alexa Fluor 488 conjugated goat anti-mouse polyclonal antibody (Abcam; ab150113) and Alexa Fluor 594 conjugated goat anti-rabbit polyclonal antibody (Abcam; ab150080). The nuclear DNA was labeled with 4′,6-diamidino-2-phenylindole solution (Beyotime, Shanghai, China). Finally, all the cells were visualized with a Leica DM-IRE2 confocal microscope using a 63× immersion oil objective. Images were captured with the Leica Application Suite advanced fluorescence software (LAS X) and the ImageJ package.

### Western Blot

Cells were lysed by radio immunoprecipitation assay lysis buffer (EpiZyme PC101) followed by centrifuging at 13 000 × g for 15 min at 4°C. Total protein was collected and stored at −80°C until use. The protein samples were used for sodium dodecyl sulfate polyacrylamide gel electrophoresis (SDS-PAGE) and transferred to a nitrocellulose membrane that was blocked for 15 min with QuickBlock Blocking Buffer for Western Blot (Beyotime, Shanghai, China). They were then incubated overnight at 4°C with primary antibodies including: rabbit polyclonal antibody to IFITM1, IFITM2, IFITM3 (Proteintech; 11727-3-AP, 12769-1-AP, 11714-1-AP), IFITM5 (Abcam; ab230863) and β-actin (CST; 4970S), anti-ASFV p30 mouse monoclonal antibody (provided by our laboratory) and anti-ASFV p72 mouse monoclonal antibody (Zoonogen M100068), followed by a 1 h incubation with secondary antibodies: Alexa Fluor 790 conjugated goat anti-mouse polyclonal antibody (Abcam; ab186695) and Alexa Fluor 680 conjugated goat anti-rabbit polyclonal antibody (Abcam: ab186696). Protein bands were visualized by Odyssey Sa (LI-COR, Lincoln, NE, USA). WB quantification by densitometry for each viral proteins or SwIFITM proteins and beta-actin are expressed using the ImageJ package.

### HAD Assay

The ASFV was quantified using the hemadsorption (HAD) assay as previously described (21). PAMs were seeded in 96-well plates with 1% pig blood cells. Viral samples were then inoculated into PAMs and titrated in triplicate using 10-fold serial dilutions. Cultures were observed for Hemadsorption (HAD) phenomena on day 7 post-infection. The HAD_50_/mL values were calculated by using the Reed and Muench method (22).

### Cell Proliferation Assay

The CCK-8 Cell Counting Kit (Vazyme, Nanjing, China) was used to determine the proliferation of WT, control or csIFITMs^−^ Vero cells. Briefly, the three Vero cells were distributed into 96-well plates, and their proliferation was then detected every 12 h. Besides, the WT and csIFITMs^−^ Vero cells were transiently transfected with five pEF-SwIFITMs or empty plasmid pEF6/Myc-His for 24 h and then infected with ASFV for 36 h; their proliferation was detected every 6 hours after transfection. The experiments were included with three replicates and non-transfected blank and empty plasmid control was included. CCK8 was added and incubated for 1 h; the absorbance was measured at 450 nm.

### Statistical Analysis

GraphPad Prism v6.02 was used for all statistical significance calculations using the conventional Student’s *t*-test. Values are presented as the mean ± SD of three independent experiments. *P*-values of <0.05 were considered to indicate statistical significance (^*^ *p*<0.05; ^**^ *p*<0.01; ^***^ *p*<0.001; ^****^ *p*<0.0001).

## Results

### Expression Levels of SwIFITMs

**Figure 1:**
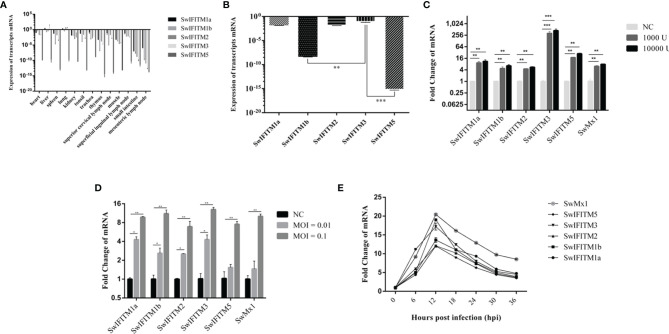
The expression level of SwIFITMs. **(A)** Expression levels of SwIFITMs in different porcine tissues were normalized by β-actin mRNA. **(B)** Expression levels of SwIFITMs in PAMs were normalized by β-actin mRNA and SwIFITM3 served as a control. **(C)** Expression levels of SwIFITMs in PAMs after treatment with IFN-α for 24 h were compared with untreated PAM mRNA, Mx1 served as a control. **(D)** Expression levels of SwIFITMs in PAMs after being infected with ASFV for 24 h were compared with untreated PAM mRNA. **(E)** Expression levels of SwIFITMs in PAMs after being infected with ASFV (0.1 MOI) each 6 h from 0 to 36 hpi. All samples were analyzed in triplicate, and three independent experiments were performed. ^*^ *p*<0.05; ^**^ *p*<0.01; ^***^ *p*<0.001 vs negative control group. Error bars indicate standard deviation.

To understand expression levels of SwIFITMs in different porcine tissues, we collected 13 tissue samples (heart, liver, spleen, lung, kidney, tonsil, trachea, thymus, superior cervical lymph node, muscle, superficial inguinal lymph node, small intestine, and mesenteric lymph node), and we then quantified their IFITM expression levels by RT-qPCR (**Figure 1A**). In this study, SwIFITMs were constitutively expressed in most porcine tissues, and the expression levels of SwIFITM1a, 1b, 2, and 3 were the highest in the liver or lung and the lowest in the small intestine. SwIFITM5 was expressed at a low level in each tissue. Moreover, the expression levels of SwIFITMs were not constant between different tissues. RT-qPCR results showed that most SwIFITMs had an expression level of >10^−5^-fold that of β-actin, except thymus SwIFITM1b and all of SwIFITM5.

In PAMs (**Figure 1B**), SwIFITM1a, -2, and -3 had an expression level of 10^−3^–10^−2^ fold that of β-actin. The expression levels of SwIFITM1a and -2 were equal to that of SwIFITM3, and the expression levels of SwIFITM1b and -5 were significantly lower than that of SwIFITM3 (*p*<0.01). Then, we estimated the expression level of SwIFITMs *in vitro* when PAMs were treated with recombinant porcine interferon-α (Novus, IFN-α) (1000 U, 10 000 U) (**Figure 1C**). As a control, addition of IFN-α caused an increase in the expression of swine Mx1 in PAMs. The expression of all SwIFITMs was up-regulated (*p*<0.001). Interestingly, it was observed that IFN-α induced the highest up-regulation of about 256-fold the SwIFITM3 expression. Next, we investigated the expression of SwIFITMs *in vitro* when PAMs were infected with ASFV (GZ201801, 0.01, 0.1 MOI) (**Figure 1D**). At 24 hpi, ASFV caused an increase in the expression of all SwIFITMs (*p*<0.01). To assess when the expression of SwIFITMs triggered in PAMs during infection of ASFV (0.1 MOI), we determined the expression of SwIFITMs each 6 h from 0 to 36 hpi (**Figure 1E**). The expression of all SwIFITMs was ascended as early as 6 hpi and reached the peak at 12 hpi which indicated that the expression of SwIFITMs was up-regulated before ASFV had achieved replication.

### Subcellular Localization of SwIFITM Proteins

**Figure 2:**
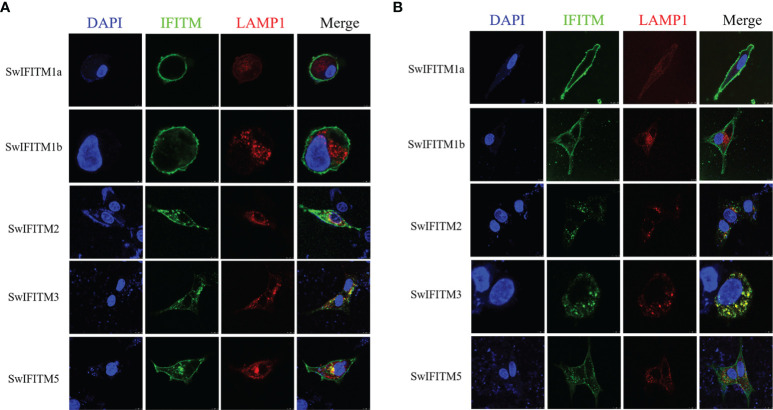
Subcellular localization of SwIFITMs in PK-15 cells and Vero cells. The cell nucleus, expression of SwIFITM1a, -1b, -2, -3, and -5 proteins, and LAMP1-positive late endosomes (red) in **(A)** PK-15 cells and **(B)** Vero cells are indicated by blue, green, and red, respectively.

To determine the subcellular localization of SwIFITMs, PK-15 cells and Vero cells were transiently transfected with five pEF-SwIFITMs including an N-terminal FLAG tag for 24 h, and they were then evaluated by confocal immunofluorescence microscopy (**Figures 2A, B** **)**. In PK-15 cells, it was revealed that SwIFITM1a and -1b localized predominantly to the plasma membrane, but SwIFITM3 mainly focused on the cytoplasm and the perinuclear region, otherwise SwIFITM2 and -5 accumulated in the plasma membrane and the cytoplasm. Confocal microscopy revealed that only SwIFITM3 and -5 were colocalized with LAMP1 in late endosomal compartments in PK-15 cells. In Vero cells, it was observed that SwIFITM1a and -1b localized predominantly to cell surface similar to PK-15 cells, whereas SwIFITM2 and -3 was present the cytoplasm and the perinuclear region, and SwIFITM5 localized in the cell surface and the cytoplasm. SwIFITM3 and -5 had colocalization with LAMP1, but the amount of colocality of SwIFITM5 and LAMP1 in Vero cells was significantly lower than that in PK-15 cells. SwIFITMs localized to distinct cellular compartments.

### Knockdown of SwIFITMs Enhances ASFV Replication

**Figure 3:**
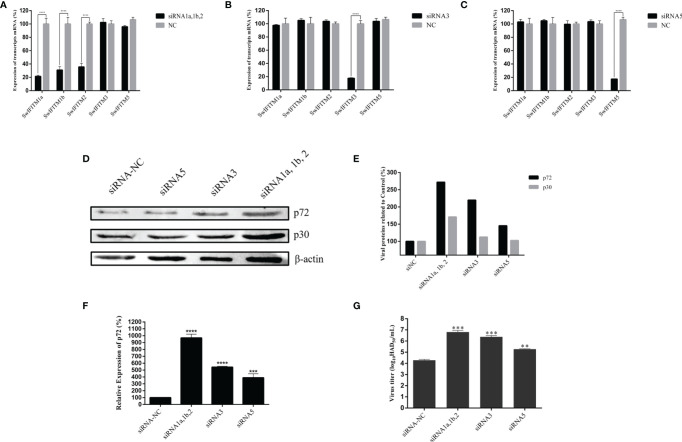
Knockdown of SwIFITMs enhances ASFV replication. **(A)** SwIFITM mRNA expression levels were evaluated in PAMs 24 h after transfection of 50 nmol of siRNA1a, 1b, 2, or negative control. **(B)** SwIFITM mRNA expression levels were evaluated in PAMs 24 h after transfection of 50 nmol of siRNA3 or negative control. **(C)** SwIFITM mRNA expression levels were evaluated in PAMs 24 h after transfection of 50 nmol of siRNA5 or negative control. siRNAs were transfected into PAMs separately for 24 h and then infected with 0.1 MOI of ASFV; the virus replication level of ASFV was determined by **(D)** RT-qPCR and **(E)** western blotting with β-actin used as a control at 36 hpi; **(F)** WB quantification by densitometry for each viral protein and β-actin, and the density quantitative ratios of each viral protein and β-actin were calculated; **(G)** the viral titration of progeny virus was tested in HAD assay. Samples were analyzed in triplicate, and three independent experiments were performed. ^**^ *p* < 0.01; ^***^ *p*<0.001; ^****^ *p*<0.0001 vs negative control group. Error bars indicate standard deviation.

To primarily explore the effect of SwIFITMs on ASFV replication, we designed and synthesized three different siRNAs to knockdown endogenous SwIFITMs. Because of the high nucleotide-sequence homologies of SwIFITM1a, -1b and -2, we designed a single siRNA targeting all three of them. The siRNAs were transfected into PAMs separately for 24 h (**Figures 3A–C**) and then infected with ASFV. At 36 hpi, all samples were harvested to detect their virus replication levels by RT-qPCR and WB **(** **Figures 3D–F** **)** using an ASFV p72 probe, anti-ASFV p30 polyclonal antibody and anti-ASFV p72 polyclonal antibody, besides, the viral titration of progeny virus was tested in HAD assay **(** **Figure 3G** **)**. The results indicated that the parameters relating to ASFV replication in siIFITM1a,1b,2, siIFITM3 and siIFITM5 were significantly increased compared with the siNC group. Although siIFITM1a,1b,2 caused the highest replication levels of ASFV, further study is needed to verify the relationship between these three genes and ASFV restriction because, as noted, siRNA1a,1b,2 jointly targeted SwIFITM1a, -1b, and -2.

### Overexpression of SwIFITMs Inhibits ASFV Replication

**Figure 4:**
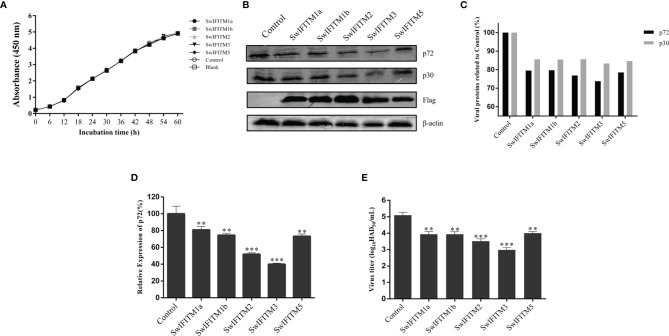
Overexpression of SwIFITMs Inhibits ASFV replication. pEF-SwIFITMs with FLAG tags were transfected into WT Vero cells separately for 24 h and then infected with 0.1 MOI of ASFV; **(A)** the cells viability was detected by cell proliferation assay; **(B, C)** the virus replication levels of ASFV were determined by western blotting at 36 hpi, and band densitometry for each protein was determined, and the density quantitative ratios of each viral or SwIFITM protein and β-actin were calculated, ratio of SwIFITM protein and β-actin served as a control; **(D)** the expression levels of p72 protein were detected by RT-qPCR with β-actin used as a control at 36 hpi; **(E)** the viral titration of progeny virus was tested in HAD assay. Samples were analyzed in triplicate, and three independent experiments were performed. ^**^ *p*<0.01; ^***^ *p*<0.001; vs empty vector control group. Error bars indicate standard deviation.

Next, we analyzed the impact of SwIFITM overexpression on ASFV replication. Vero cells were transiently transfected with five pEF-SwIFITMs for 24 h and then infected with ASFV. At 36 hpi, all samples were collected to detect the cells proliferation assay and their virus replication levels by RT-qPCR, WB and HAD assay (**Figures 4A–E**). The results of the cells proliferation assays indicated that the cells viability in transfected cells with the different SwIFITM were not significantly different from that of the empty plasmid control or non-transfected cells. Compared with control infection, the viral production of ASFV in the five overexpression experimental groups was significantly decreased, indicating that SwIFITM1a, -1b, -2, -3, and -5 can restrict ASFV replication. The HAD assay results further verified that all the SwIFITMs overexpression reduced ASFV replication.

### Generation and Validation of csIFITM Knockout Vero Cell Lines

**Figure 5:**
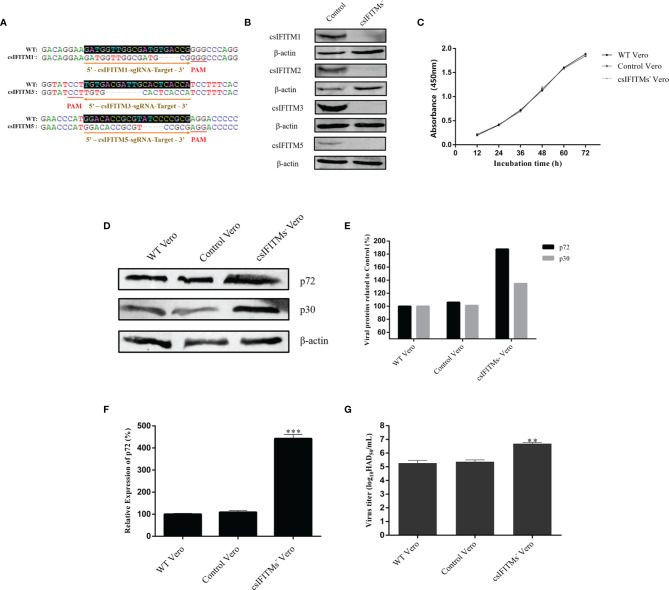
Generation and validation of csIFITM knockout Vero cell lines. **(A)** Three different sgRNA targeting sites and the result of sanger sequencing with WT and csIFITMs^−^ Vero cells. **(B)** Expression of endogenous csIFITMs was detected by western blotting with β-actin used as a control. **(C)** Proliferation of csIFITMs^−^, control, and untreated Vero cells were detected by the CCK-8 Cell Counting method. The csIFITMs^−^, control, and untreated Vero cells were infected with 0.1 MOI of ASFV; **(D, E)** the virus replication levels of ASFV were determined by western blotting at 36 hpi, and band densitometry for each protein was determined, and the density quantitative ratios of each viral or SwIFITM protein and β-actin were calculated, ratio of SwIFITM protein and β-actin served as a control; **(F)** the expression levels of p72 protein were detected by RT-qPCR with β-actin used as a control at 36 hpi; **(G)** the viral titration of progeny virus was tested in HAD assay. Samples were analyzed in triplicate, and three independent experiments were performed. ^**^ *p* < 0.01; ^***^ *p*<0.001 vs empty vector control group. Error bars indicate standard deviation.

Because Vero cells constitutively express endogenous IFITMs that could interfere with subsequent experiments, it is necessary to construct knockout Vero cell lines that block endogenous IFITMs. Three different sgRNAs targeting the csIFITM1, -3, and -5 first exons were designed and inserted into the lentiviral vector lentiCRISPRv2-DsRed2 to generate lentiCRISPRv2-DsRed2-csIFITMs. These were used to establish the knockout cell lines by a lentiviral transduction system and were screened by limiting dilution assay. Once the csIFITMs^−^ Vero cells were established., the knockout of csIFITMs was determined by Sanger sequencing validation or WB analysis (**Figures 5A, B** **)**. The Sanger sequencing results showed that csIFITM1, -3, and -5 respectively had 4, 7, and 5 bp deletion mutations in the csIFITMs^−^ Vero cells. The WB results further verified that the csIFITM genes in the Vero cells were successfully knocked out, and the csIFITMs^−^ Vero cell line was obtained.

Interestingly, due to the failure to successfully find and amplify csIFITM2, we did not attempt to knock out IFITM2 at the same time, but the WB results showed that csIFITM2 was nonetheless knocked out. This indicates that csIFITM2 may be highly homologous with csIFITM3, resulting in its simultaneous knockout. Next, we detected the proliferation of the csIFITMs^−^, control, and WT Vero cells using the CCK-8 Cell Counting Kit (**Figure 5C**). The results showed that the proliferation of the csIFITMs^−^ Vero cells was not significantly different from that of the control and WT Vero cells, suggesting that the knockout of csIFITMs did not affect cell viability. Finally, the three types of cells were infected with ASFV. All samples were collected to detect their virus replication levels by RT-qPCR, WB and HAD assay at 36 hpi (**Figures 5D–G**). The results indicated that the viral production of ASFV in csIFITMs− Vero cells was significantly increased: more than 10-fold when compared with the control and WT Vero cells.

### Antiviral Effect of SwIFITMs on ASFV

**Figure 6:**
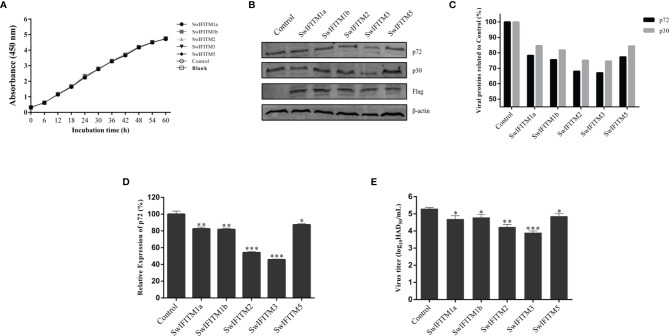
Antiviral effect of SwIFITMs on ASFV. pEF-SwIFITMs with FLAG stag were transfected into csIFITMs^−^ Vero cells separately for 24 h and then infected with 0.1 MOI of ASFV; **(A)** the cells viability was detected by cell proliferation assay; **(B, C)** the virus replication levels of ASFV were determined by western blotting at 36 hpi, and band densitometry for each protein was determined, and the density quantitative ratios of each viral or SwIFITM protein and β-actin were calculated, ratio of SwIFITM protein and β-actin served as a control; **(D)** the expression levels of p72 protein were detected by RT-qPCR with β-actin used as a control at 36 hpi; **(E)** the viral titration of progeny virus was tested in HAD assay. Samples were analyzed in triplicate, and three independent experiments were performed. ^*^ *p* < 0.05; ^**^ *p* < 0.01; ^***^ *p*<0.001 vs empty vector control group. Error bars indicate standard deviation.

To analyze the antiviral effect of SwIFITMs on ASFV infection in csIFITMs^−^ Vero cells, csIFITMs^−^ Vero cells were transiently transfected with five pEF-SwIFITMs for 24 h, and they were then infected with ASFV. At 36 hpi, all samples were collected to detect the cells proliferation assays and their virus replication levels by RT-qPCR, WB and HAD assay (**Figures 6A–E**). The results of the cells proliferation assay indicated that there was no significant difference in cell viability under all conditions. Compared with control infection, the overexpression of SwIFITM1a, -1b, -2, -3, or -5 restricted the replication of ASFV. The HAD assay results were further confirmed that each of the SwIFITMs overexpression restricted ASFV replication.

## Discussion

After infection with pathogens such as bacteria or viruses, host cells activate innate immune responses as their first line of defense. As downstream effector molecules in the natural immune system, natural immune factors are an important part of the antiviral natural immune system (23, 24). They have a wide variety—there are more than 1000—and they have a variety of strategies to limit virus replication. The cytokine group known as IFN encodes a variety of natural antiviral immune effectors through the induction cascade of ISG. Among these, IFITM is an important protein in the CD225 protein superfamily that is intensely induced during viral infection, and it is widely distributed in animal cells (25). SwIFITMs have been shown to widely inhibit viral infection, especially in RNA viruses like porcine reproductive and respiratory syndrome virus, classical swine fever virus, and IAV (26–28).

As the only known DNA arbovirus, ASFV has strong environmental resistance and a wide range of transmission routes. As such, it causes significant economic losses to the global porcine industry (19, 29). However, there have as yet been no reports of studies on the antiviral effects of SwIFITMs on ASFV. In this work, we verified the SwIFITM expression patterns in different porcine tissues and PAMs, as well as the subcellular localization in PK-15 cells, and we identified its antiviral activity against ASFV in PAMs and Vero cells.

Because most vertebrate IFITM families contain multiple IFITM homologues (25, 30), we successfully amplified SwIFITM1a, -1b, -2, -3, and -5 which contained the same key amino acid sites and functional domains (13). We then analyzed the expression levels of SwIFITMs, and the results indicated that SwIFITMs are constitutively expressed in most porcine tissues. We also found that IFN treatment or virus infection could induce SwIFITM expression in PAMs.

The subcellular localization of SwIFITM proteins in PK-15 cells and Vero cells, and its localization patterns associated with endocytic compartments, especially late endosomal compartments, were partly coincident with previous reports. The results revealed that SwIFITM1a and -1b localized predominantly to the plasma membrane, but SwIFITM3 mainly focused on the cytoplasm and the perinuclear region, which is mostly similar to the distribution of their human homologues. We also identified that SwIFITM2 and -5 accumulated in the cell surface and cytoplasm. In a previous study, swine and human SwIFITM2 and -3 were found to share an N-terminal extension of about 20 amino acids that contains the endocytosis motif YEML, and all the SwIFITM1a, -1b and -5 lack the same motif structure (31, 32). Interestingly, we also found that the overexpression of SwIFITM5 was colocalized with the late endosomal marker LAMP1, which might be attributed to the alteration of the lysosome distribution by overexpression or the interaction with IFITM5 proteins and other IFITM paralogues (33, 34).

We have now determined that all five SwIFITMs have an antiviral effect on ASFV replication. The overexpression of SwIFITM1a, -1b, -2, -3, or -5 could significantly inhibit ASFV replication in Vero cells, whereas knockdown of these genes could enhance ASFV replication in PAMs. This showed that SwIFITM1a, -1b, -2, -3, and -5 can restrict ASFV replication individually, and all of them play a restrictive part in the early or late stage of ASFV replication. We blocked the constitutive expression of endogenous IFITMs in Vero cells using a CRISPR-Cas9 system and then infected them with ASFV. The results indicated that the knockout of endogenous IFITMs could enhance ASFV replication (9, 35). Finally, we analyzed the antiviral effect of SwIFITMs on ASFV infection in csIFITMs^−^ Vero cells. The overexpression of SwIFITM1a, -1b, -2, -3, and -5 was found to potently restrict ASFV replication, which was consistent with the previous experimental results in wild-type Vero cells.

The GZ201801 strain of ASFV we used is a non-adapted field isolate which replication is not supported in Vero cells. However, our results may be contrary to this conclusion. In this study, we used the GZ201801 strain to infect Vero cells, the virus titres could reach more than 10^5^ HAD_50_/mL at 36 hpi. Perhaps the virus has mutated during Vero cell passage and produced adaptability. In addition, it is also possible that the Vero cells we used are different from those used in other laboratories, resulting in mutations in the culture process, which can make the strain adapt to growth. African green monkey cells MA-104 has been showed to be suitable for ASFV field isolate (36). Similarly, it may not be uncommon for the GZ201801 strain to adapt in Vero cells which are also African green monkey cells.

SwIFITM proteins play different antiviral roles due to their different distribution and expression in cells. Given that ASFV entry is strongly dependent on endocytosis, SwIFITM1a and -1b may restrict the fusion of ASFV membrane and plasma membrane, and then inhibit viral entry (37). SwIFITM2 and -3 may restrict ASFV entry and uncoating, and inhibit the release of virus in late endosomes or lysosomes (38, 39). Since little is known about the antiviral mechanism of IFITM5, SwIFITM5 may play its antiviral role by interacting with other homologues, but further research is still needed to verify it.

This study verifies that SwIFITMs are a group of antiviral restriction factors, which means it may be possible to develop them as antiviral drugs or vaccine targets. Although there is still a need to determine the antiviral functions and molecular mechanisms of SwIFITMs against ASFV, our research will further expand our understanding of the anti-ASFV activity of porcine IFITM.

## Data Availability Statement

The original contributions presented in the study are included in the article/supplementary material. Further inquiries can be directed to the corresponding authors.

## Ethics Statement

The animal study was reviewed and approved by the South China Agricultural University Experimental Animal Welfare Ethics Committee.

## Author Contributions

Conceptualization: GZ and GL. Methodology: SC, ZZ, and JC. Software: SC, ZZ, JC, and LZ. Validation: RS, FZ, ZL, and JO. Formal Analysis: SC and ZZ. Investigation: SC and LX. Resources: SC and GL. Writing – Original Draft Preparation: SC. Writing – Review & Editing: GZ and GL. Visualization: SC. Supervision: PZ and GZ. Project Administration: PZ and GZ. Funding Acquisition: GZ. All authors contributed to the article and approved the submitted version.

## Funding

This research was funded by the Key-Area Research and Development Program of Guangdong Province [grant number 2019B020211003], National Natural Science Foundation of China [grant number 31941004], The National Key Research and Development Program of China (2021YFD1800104), Independent Research and Development Projects of Maoming Laboratory (2021ZZ003) and China Agriculture Research System of MOF and MARA (cars-35).

## Conflict of Interest

The authors declare that the research was conducted in the absence of any commercial or financial relationships that could be construed as a potential conflict of interest.

## Publisher’s Note

All claims expressed in this article are solely those of the authors and do not necessarily represent those of their affiliated organizations, or those of the publisher, the editors and the reviewers. Any product that may be evaluated in this article, or claim that may be made by its manufacturer, is not guaranteed or endorsed by the publisher.

## References

[1] Brass AL Huang IC Benita Y John SP Krishnan MN Feeley EM. The IFITM Proteins Mediate Cellular Resistance to Influenza A H1N1 Virus, West Nile Virus, and Dengue Virus. Cell (2009) 139(7):1243–54. doi: 10.1016/j.cell.2009.12.017 PMC2824905 20064371

[2] Li LF Yu J Zhang Y Yang Q Li Y Zhang L. Interferon-Inducible Oligoadenylate Synthetase-Like Protein Acts as an Antiviral Effector Against Classical Swine Fever Virus *via* the MDA5-Mediated Type I Interferon-Signaling Pathway. J Virol (2017) 91(11):1514–6. doi: 10.1128/JVI.01514-16 PMC5432864 28331099

[3] Chan YK Huang IC Farzan M. IFITM Proteins Restrict Antibody-Dependent Enhancement of Dengue Virus Infection. PloS One (2012) 7(3):e34508. doi: 10.1371/journal.pone.0034508 22479637 PMC3316688

[4] Desai TM Marin M Chin CR Savidis G Brass AL Melikyan GB. IFITM3 Restricts Influenza A Virus Entry by Blocking the Formation of Fusion Pores Following Virus-Endosome Hemifusion. PloS Pathog (2014) 10(4):e1004048. doi: 10.1371/journal.ppat.1004048 24699674 PMC3974867

[5] Narayana SK Helbig KJ McCartney EM Eyre NS Bull RA Eltahla A. The Interferon-Induced Transmembrane Proteins, IFITM1, IFITM2, and IFITM3 Inhibit Hepatitis C Virus Entry. Biol Chem (2015) 290:25946–59. doi: 10.1074/jbc.M115.657346 PMC4646249 26354436

[6] Wrensch F Karsten CB Gnirss K Hoffmann M Lu K Takada A. Interferon-Induced Transmembrane Protein-Mediated Inhibition of Host Cell Entry of Ebolaviruses. Infect Dis (2015) 212(Suppl 2):S210–8. doi: 10.1093/infdis/jiv255 PMC4564551 26034199

[7] Zhu R Wang J Lei XY Gui JF Zhang QY. Evidence for Paralichthys Olivaceus IFITM1 Antiviral Effect by Impeding Viral Entry Into Target Cells. Fish Shellfish Immunol (2013) 35(3):918–26. doi: 10.1016/j.fsi.2013.07.002 PMC7128638 23850425

[8] Smith SE Gibson MS Wash RS Ferrara F Wright E Temperton N. Chicken Interferon-Inducible Transmembrane Protein 3 Restricts Influenza Viruses and Lyssaviruses *In Vitro* . J Virol (2013) 87(23):12957–66. doi: 10.1128/JVI.01443-13 PMC3838109 24067955

[9] Muñoz-Moreno R Cuesta-Geijo MÁ Martínez-Romero C Barrado-Gil L Galindo I García-Sastre A. Antiviral Role of IFITM Proteins in African Swine Fever Virus Infection. PloS One (2016) 11(4):e0154366. doi: 10.1371/journal.pone.0154366 27116236 PMC4846163

[10] Ivanovic T Choi JL Whelan SP van Oijen AM Harrison SC. Influenza-Virus Membrane Fusion by Cooperative Fold-Back of Stochastically Induced Hemagglutinin Intermediates. eLife (2013) 2:e00333. doi: 10.7554/eLife.00333 23550179 PMC3578201

[11] Tartour K Nguyen XN Appourchaux R Assil S Barateau V Bloyet LM. Interference With the Production of Infectious Viral Particles and Bimodal Inhibition of Replication Are Broadly Conserved Antiviral Properties of IFITMs. PloS Pathog (2017) 13(9):e1006610. doi: 10.1371/journal.ppat.1006610 28957419 PMC5619827

[12] Liao Y Goraya MU Yuan X Zhang B Chiu SH Chen JL. Functional Involvement of Interferon-Inducible Transmembrane Proteins in Antiviral Immunity. Front Microbiol (2019) 10:1097. doi: 10.3389/fmicb.2019.01097 31156602 PMC6532022

[13] Lanz C Yanguez E Andenmatten D Stertz S. Swine Interferon-Inducible Transmembrane Proteins Potently Inhibit Influenza A Virus Replication. J Virol (2015) 89(1):863–9. doi: 10.1128/JVI.02516-14 PMC4301123 25320322

[14] Diamond MS Farzan M. The Broad-Spectrum Antiviral Functions of IFIT and IFITM Proteins. Nat Rev Immunol (2013) 13(1):46–57. doi: 10.1038/nri3344 23237964 PMC3773942

[15] Huang IC Bailey CC Weyer JL Radoshitzky SR Becker MM Chiang JJ. Distinct Patterns of IFITM-Mediated Restriction of Filoviruses, SARS Coronavirus, and Influenza A Virus. PloS Pathog (2011) 7(1):e1001258. doi: 10.1371/journal.ppat.1001258 21253575 PMC3017121

[16] John SP Chin CR Perreira JM Feeley EM Aker AM Savidis G. The CD225 Domain of IFITM3 Is Required for Both IFITM Protein Association and Inhibition of Influenza A Virus and Dengue Virus Replication. J Virol (2013) 87(14):7837–52. doi: 10.1128/JVI.00481-13 PMC3700195 23658454

[17] Lu J Pan Q Rong LW He W Liu SL Liang C. The IFITM Proteins Inhibit HIV-1 Infection. J Virol (2011) 85(5):2126–37. doi: 10.1128/JVI.01531-10 PMC3067758 21177806

[18] Alonso C Borca M Dixon L Revilla Y Rodriguez F Escribano JM. ICTV Virus Taxonomy Profile: Asfarviridae. J Gen Virol (2018) 99:613–4. doi: 10.1099/jgv.0.001049 PMC12662184 29565243

[19] Dixon LK Sun H Roberts H. African Swine Fever. Antiviral Res (2019) 165:34–41. doi: 10.1016/j.antiviral.2019.02.018 30836106

[20] Shalem O Sanjana NE Hartenian E Xi S Scott DA Tarjei M. Genome-Scale CRISPR-Cas9 Knockout Screening in Human Cells. Science (2014) 343(6166):84–7. doi: 10.1126/science.1247005 PMC4089965 24336571

[21] Malmquist WA Hay D. Hemadsorption and Cytopathic Effect Produced by African Swine Fever Virus in Swine Bone Marrow and Buffy Coat Cultures. Am J Vet Res (1960) 21:104–8. 14420403

[22] Reed LJ Muench H. A Simple Method of Estimating Fifty Percent Endpoints. Am J Hyg (1938) 27:493–7. doi: 10.1093/oxfordjournals.aje.a118408

[23] Anafu AA Bowen CH Chin CR Brass AL Holm GH. Interferon-Inducible Transmembrane Protein 3 (IFITM3) Restricts Reovirus Cell Entry. J Biol Chem (2013) 288(24):17261–71. doi: 10.1074/jbc.M112.438515 PMC3682530 23649619

[24] Correia S Ventura S Parkhouse RM. Identification and Utility of Innate Immune System Evasion Mechanisms of ASFV. Virus Res (2013) 173(1):87–100. doi: 10.1016/j.virusres.2012.10.013 23165138

[25] Sällman Almén M Bringeland N Fredriksson R Schiöth HB. The Dispanins: A Novel Gene Family of Ancient Origin That Contains 14 Human Members. PloS One (2012) 7(2):e31961. doi: 10.1371/journal.pone.0031961 22363774 PMC3282796

[26] Li C Zheng HQ Wang YF Dong W Liu YR Zhang L. Antiviral Role of IFITM Proteins in Classical Swine Fever Virus Infection. Viruses (2019) 11(2):126. doi: 10.3390/v11020126 PMC6409519 30704088

[27] Lin TY Chin CR Everitt AR Clare S Perreira JM Savidis G. Amphotericin B Increases Influenza A Virus Infection by Preventing IFITM3-Mediated Restriction. Cell Rep (2013) 5(4):895–908. doi: 10.1016/j.celrep.2013.10.033 24268777 PMC3898084

[28] Zhang A Duan H Zhao HJ Liao HC Du YK Li LL. Interferon-Induced Transmembrane Protein 3 Is a Virus-Associated Protein Which Suppresses Porcine Reproductive and Respiratory Syndrome Virus Replication by Blocking Viral Membrane Fusion. J Virol (2020) 94(24):e01350–20. doi: 10.1128/JVI.01350-20 PMC7925183 32999030

[29] Gaudreault NN Madden DW Wilson WC Trujillo JD Richt JA. African Swine Fever Virus: An Emerging DNA Arbovirus. Front Vet Sci (2020) 7:215. doi: 10.3389/fvets.2020.00215 32478103 PMC7237725

[30] Melvin WJ McMichael TM Chesarino NM Hach JC Yount JS. IFITMs From Mycobacteria Confer Resistance to Influenza Virus When Expressed in Human Cells. Viruses (2015) 7(6):3035–52. doi: 10.3390/v7062759 PMC4488726 26075508

[31] Chesarino NM McMichael TM Hach JC Yount JS. Phosphorylation of the Antiviral Protein Interferon-Inducible Transmembrane Protein 3 (IFITM3) Dually Regulates its Endocytosis and Ubiquitination. J Biol Chem (2014) 289(17):11986–92. doi: 10.1074/jbc.M114.557694 PMC4002105 24627473

[32] Jia R Xu F Qian J Yao Y Miao C Zheng YM. Identification of an Endocytic Signal Essential for the Antiviral Action of IFITM3. Cell Microbiol (2014) 16(7):1080–93. doi: 10.1111/cmi.12262 PMC4065222 24521078

[33] Liu PC Zhang YT Zhang SS Peng CM Yang W Li XX. Integrative Overview of IFITMs Family Based On Bioinformatics Analysis. Intractable Rare Dis Res (2021) 10(3):165–72. doi: 10.5582/irdr.2021.01041 PMC8397817 34466338

[34] Winkler M Wrensch F Bosch P Knoth M Schindler M Gärtner S. Analysis of IFITM-IFITM Interactions by a Flow Cytometry-Based FRET Assay. Int J Mol Sci (2019) 20(16):3859. doi: 10.3390/ijms20163859 PMC6719045 31398796

[35] Qian J Le DY Wang YM Pan QH Ding SL Zheng YM. Primate Lentiviruses Are Differentially Inhibited by Interferon-Induced Transmembrane Proteins. J Virol (2015) 474:10–8. doi: 10.1016/j.virol.2014.10.015 PMC4581848 25463599

[36] Rai A Pruitt S Ramirez-M E Vuono EA Silva E Velazquez-Salinas L. Identification of a Continuously Stable and Commercially Available Cell Line for the Identification of Infectious African Swine Fever Virus in Clinical Samples. Viruses (2020) 12(8):820. doi: 10.3390/v12080820 PMC7472077 32731642

[37] Bailey CC Zhong G Huang IC Farzan M. IFITM-Family Proteins: The Cell’s First Line of Antiviral Defense. Annu Rev Virol (2014) 1:261–83. doi: 10.1146/annurev-virology-031413-085537 PMC4295558 25599080

[38] Li C Du SW Tian MY Wang YH Bai JY Tan P. The Host Restriction Factor Interferon-Inducible Transmembrane Protein 3 Inhibits Vaccinia Virus Infection. Front Immunol (2018) 9:228. doi: 10.3389/fimmu.2018.00228 29503647 PMC5820317

[39] Feeley EM Sims JS John SP Chin CR Pertel T Chen LM. IFITM3 Inhibits Influenza A Virus Infection by Preventing Cytosolic Entry. PloS Pathog (2011) 7(10):e1002337. doi: 10.1371/journal.ppat.1002337 22046135 PMC3203188

